# Rolling Circle Translation of Circular RNA in Living Human Cells

**DOI:** 10.1038/srep16435

**Published:** 2015-11-10

**Authors:** Naoko Abe, Ken Matsumoto, Mizuki Nishihara, Yukiko Nakano, Aya Shibata, Hideto Maruyama, Satoshi Shuto, Akira Matsuda, Minoru Yoshida, Yoshihiro Ito, Hiroshi Abe

**Affiliations:** 1 Department of Chemistry, Graduate School of Science, Nagoya University, Furo-cho, Chikusa-ku, Nagoya 464-8602, Japan; 2 Faculty of Pharmaceutical Sciences, Hokkaido University, Kita-12, Nishi-6, Kita-ku, Sapporo 060-0812, Japan; 3 Nano Medical Engineering laboratory, RIKEN, 2-1 Hirosawa, Wako-Shi, Saitama 351-0198, Japan; 4 PRESTO, Japan Science and Technology Agency, 4-1-8 Honcho, Kawaguchi, Saitama 332-0012, Japan; 5 Chemical Genetics Laboratory, RIKEN, 2-1 Hirosawa, Wako-Shi, Saitama 351-0198, Japan; 6 Emergent Bioengineering Materials Research Team, RIKEN Center for Emergent Matter Science, 2-1 Hirosawa, Wako, Saitama 351-0198, Japan

## Abstract

We recently reported that circular RNA is efficiently translated by a rolling circle amplification (RCA) mechanism in a cell-free *Escherichia coli* translation system. Recent studies have shown that circular RNAs composed of exonic sequences are abundant in human cells. However, whether these circular RNAs can be translated into proteins within cells remains unclear. In this study, we prepared circular RNAs with an infinite open reading frame and tested their translation in eukaryotic systems. Circular RNAs were translated into long proteins in rabbit reticulocyte lysate in the absence of any particular element for internal ribosome entry, a poly-A tail, or a cap structure. The translation systems in eukaryote can accept much simpler RNA as a template for protein synthesis by cyclisation. Here, we demonstrated that the circular RNA is efficiently translated in living human cells to produce abundant protein product by RCA mechanism. These findings suggest that translation of exonic circular RNAs present in human cells is more probable than previously thought.

We recently found that circular RNA containing an infinite open reading frame (ORF) can be efficiently translated to produce proteins in an *Escherichia coli* cell-free translation system^1^, in a manner similar to rolling circle amplification (RCA) of the polymerase reaction (Fig. 1)^2^,^3^,^4^,^5^. In this translation system, the circular RNA contains no stop codon and the number of nucleotides composing the RNA is a multiple of three^1^,^6^,^7^. Thus, theoretically, the elongation process can last indefinitely once translation initiation occurs^1^,^6^,^7^. The mechanism of RCA not only provides a long-repeating peptide sequence but also enhances the productivity over a given period of time, because the ribosome does not need to bind multiple times with the RNA template, which is the rate limiting step in the reaction cycle^3^. In our case, the circular template produced translation product two orders of magnitude more efficiently than its linear counterpart^1^.

In the present study, we applied circular RNA to eukaryotic translation systems, which are more complex than those in prokaryotes^8^. Synthesis of protein via RCA has not previously been reported in living eukaryotic cells; therefore, we posed the question of whether this is possible. In 1979, circular RNA was found to bind to prokaryotic but not eukaryotic ribosomes^9^,^10^. To the best of our knowledge, translation of circular RNA in the absence of any particular element for internal ribosome entry has never been reported in eukaryotic systems^7^,^11^,^12^,^13^,^14^,^15^,^16^,^17^. In general, the majority of eukaryotic messenger RNAs (mRNAs) possess a 5′ cap structure and a 3′ poly(A) tail^8^. Eukaryotic translation initiation is normally cap-dependent, because recognition of the cap is required for assembly of the initiation complex^18^. Cap-independent translation is an alternative means of translation initiation in eukaryotes that depends on the presence of particular elements that induce internal initiation, such as an internal ribosome entry site (IRES)^7^,^19^. IRES sequences were first reported in viral RNAs, and bind to eukaryotic ribosomes when internal to the RNA. In principle, the key feature of IRES-driven translation is its 5′-end independence, rather than cap-independence.

In this study, we show for the first time that circular RNA synthesised *in vitro* can be translated in living human cells in the absence of particular elements for internal initiation. Moreover, we show that protein could be synthesised from circular RNA via the RCA mechanism in eukaryotic translation systems (Fig. 1).

## Results

**Figure 2:**
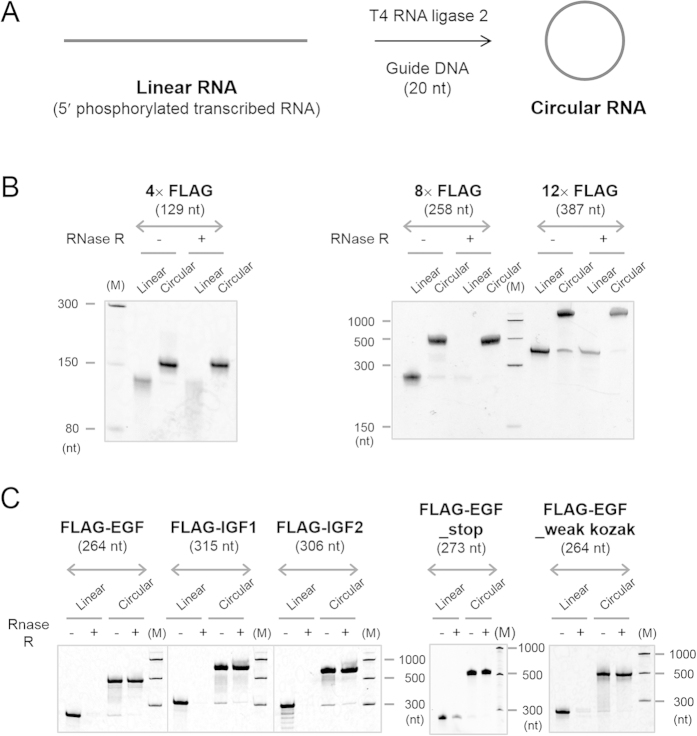
Synthesis of circular RNAs. (**A**) A scheme for the synthesis of circular RNAs used in this study. Transcribed linear RNAs were annealed to its complementary DNA oligomer and then ligated using T4 RNA ligase 2 to produce the circular RNA. (**B**,**C**) Verification of their circularity of the RNAs. The RNAs were incubated with RNase R and the reactions were analysed by denaturing PAGE. The gels were visualised by SYBR Green II staining.

**Figure 3:**
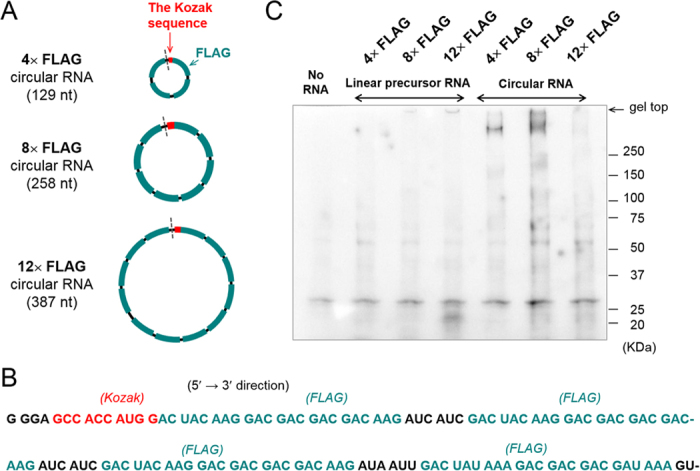
Design of circular RNAs and their translation in rabbit reticulocyte lysate (RRL). (**A**) Schematic representation of the circular RNAs used in this study. The grey dashed line represents the point of ligation of the linear RNA to form a circle. (**B**) Nucleotide sequence of **4× FLAG** circular RNA. The first nucleotide, **G**, is linked with the last nucleotide, **U**, in this context. Sequences of the other RNAs are shown in Supplementary Table S1. (**C**) Western blot analysis of the translation reaction in RRL. The three circular RNAs and their linear precursors (1 μg) were incubated at 30 °C overnight in a 25 μL reaction.

First of all, small circular RNAs of 129, 258 and 387 nucleotides, which contain multiple FLAG-coding sequences, were synthesised (Figs 2A and 3 and Supplementary Table S1). The FLAG peptide consists of eight amino acids (Asp Tyr Lys Asp Asp Asp Asp Lys), and is thus encoded by a sequence of 24 nucleotides (nt)^20^. The minimum length of the RNA circles used in this study was set as 129 nt, based on the previous finding that a circular RNA of 126 nt with multiple FLAG-coding sequences was well translated in an *E. coli* cell-free system^1^. These circular RNAs contained an infinite ORF but no particular sequence for internal initiation. In eukaryotes, the RNAs included the Kozak consensus sequence, GCCAUGG, which contained an initiation codon, and the conserved flanking nucleotides (Fig. 3)^21^. The Kozak sequence plays a major role in initiation of translation in eukaryotic systems. A circular RNA of 129 nt named **4**× **FLAG** contained four FLAG coding sequences. Larger circular RNAs of 258 nt and 387 nt, named **8×** and **12**× **FLAG**, contained eight and twelve FLAG coding sequences, respectively. AU-rich spacer sequences, such as AUCAUC, are selected between the FLAG-coding sequences to suppress undesired secondary structure formation in the RNA, which might inhibit its translation^22^. Precursor linear RNAs were transcribed *in vitro* from their dsDNA templates (Supplementary Table S2) using T7 RNA polymerase (Supplementary Fig. S1). Guanosine monophosphate was added to the reaction mixture in order to phosphorylate the transcribed RNAs at the 5′-end, which was required for the subsequent circularization reaction^7^. After purification, the linear transcribed RNAs were circularised using T4 RNA ligase 2 with a short complementary DNA sequence as the template (Fig. 2A, Supplementary Fig. S1)^7^. Circularity of synthesized RNAs were confirmed using RNase R, an 3′−5′ exoribonuclease (Fig. 2B).

In the first set of experiments, these linear and circular RNAs were tested for translation using a cell-free rabbit reticulocyte lysate system (RRL). After incubation, the reaction mixtures were subjected to sodium dodecyl sulfate polyacrylamide gel electrophoresis and western blot analysis using an anti-FLAG M2 antibody (Fig. 3C). When the **8×** and **12× FLAG** linear RNAs were incubated, bands smaller than 25 kDa were observed (Supplementary Fig. S2). To suppress the background signal, the lysates were purified using anti-FLAG M2 antibody-conjugated agarose beads prior to analysis. It is known that uncapped mRNA can also be translated by a 5′ end-dependent scanning mechanism^23^. When circular RNAs were incubated with the lysate, high-molecular weight smears could be seen just below the top of the gel, suggesting that translation had been initiated and continued on the circle^1^,^6^,^7^. Of the three RNA circles evaluated, the **8× FLAG** circular RNA produced the most high-molecular weight peptides. Assuming the molecular weight of the repeating product to be more than 300 kDa, the ribosomes would have cycled around the circle at least 15 times, based on a product of 20 kDa translated from the linear counterpart of the circular RNA (Fig. 3C and Supplementary Fig. S2)^1^.

**Figure 4:**
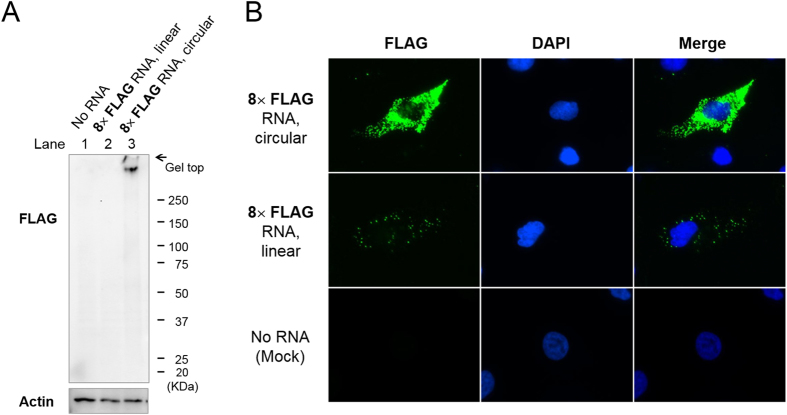
Translation of a circular 8× FLAG RNA in living HeLa cells. HeLa cells were transfected with **8× FLAG** linear or circular RNA by lipofection. (**A**) Western blot analysis of the lysate using an anti-FLAG antibody. β-Actin was detected as a loading control. (**B**) Microscopic imaging of the translation product in HeLa cells. The subcellular localisation of the FLAG-containing product was analysed by immunofluorescence staining using an anti-FLAG antibody and anti-mouse IgG antibody labelled with Alexa Fluor 488 (FLAG, green). Cells were counterstained with 4′,6-diamidino-2-phenylindole (DAPI; blue) to visualise nuclei.

Next, we exogenously introduced these circular RNAs into HeLa cells, a human epithelial cell line derived from a cervical adenocarcinoma, to determine whether they were translated. By western blot analysis of the HeLa cell lysate, FLAG-containing peptides of high-molecular weight were detected from the **8× FLAG** circular RNA (Fig. 4A). HeLa cells transfected with RNAs were then immunostained and observed by fluorescence microscopy (Fig. 4B). Fluorescent dots were visible throughout the cytoplasm of cells transfected with **8× FLAG** linear RNA, whereas no signal was detected in mock-transfected cells. More signals of higher intensity were seen in cells transfected with the circular RNA compared with the linear RNA, indicating that the amount of translation product was greater from the circular RNA. The smaller **4× FLAG** circular RNA also produced fluorescent signals in HeLa cells (Supplementary Fig. S3).

**Figure 5:**
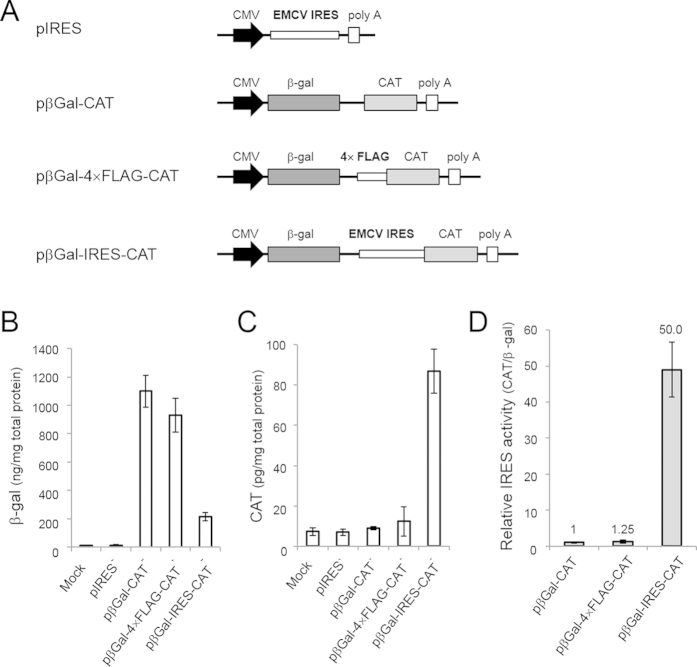
Bicistronic reporter assay for IRES activity in the repeating FLAG-coding sequence. (**A**) Schematic representation of the bicistronic plasmid constructs. pβGal–CAT contains no insert between the two cistrons, which encoded chloramphenicol acetyltransferase (CAT) and β-galactosidase (β-gal). pβGal–4× FLAG–CAT contained a repeated (four) FLAG sequence and pβGal–IRES–CAT contained an IRES sequence derived from EMCV in the region between the two cistrons. The plasmid, pIRES, was used as a negative control. (**B,C**) Expression levels of β-gal (**B**) and CAT (**C**) in the cell lysate after the transfection of these plasmids into HeLa cells. The amounts of CAT and β-gal were determined by enzyme-linked immunoabsorbent assay. Results obtained from mock-transfected control are also shown. The plotted data are the means ± standard deviation of three independent experiments. (**D**) Relative IRES activities were calculated from the data shown in (**B**,**C**). The ratio of CAT/β-gal expression for pβGal–CAT was set at 1.0.

At this point, we performed a control experiment to investigate whether the repeating FLAG-coding sequence itself possessed IRES activity (Fig. 5)^24^. We prepared a series of plasmid DNA vectors containing two reporter genes, chloramphenicol acetyltransferase (CAT) and β-galactosidase (β-gal) (Fig. 5A)^25^. To assess its IRES activity, the **4× FLAG** sequence was inserted between the two cistrons to generate the vector, pβGal–4× FLAG–CAT, and the expression of CAT from the second cistron was compared with CAT expression from a control vector without an IRES (pβGal–CAT) and a vector containing an encephalomyocarditis (EMCV) IRES (pβGal–IRES–CAT). The expression levels of β-gal and CAT are shown in Fig. 5B and C, respectively. As shown in Fig. 5D, pβGal–4× FLAG–CAT showed no substantial IRES activity, because its relative CAT/βGal expression level was comparable to that of pβGal–CAT, the control vector. Based on these results, we conclude that the translation initiation on these circular RNAs is unlikely to be attributed to an IRES activity of FLAG–coding sequences.

**Figure 6:**
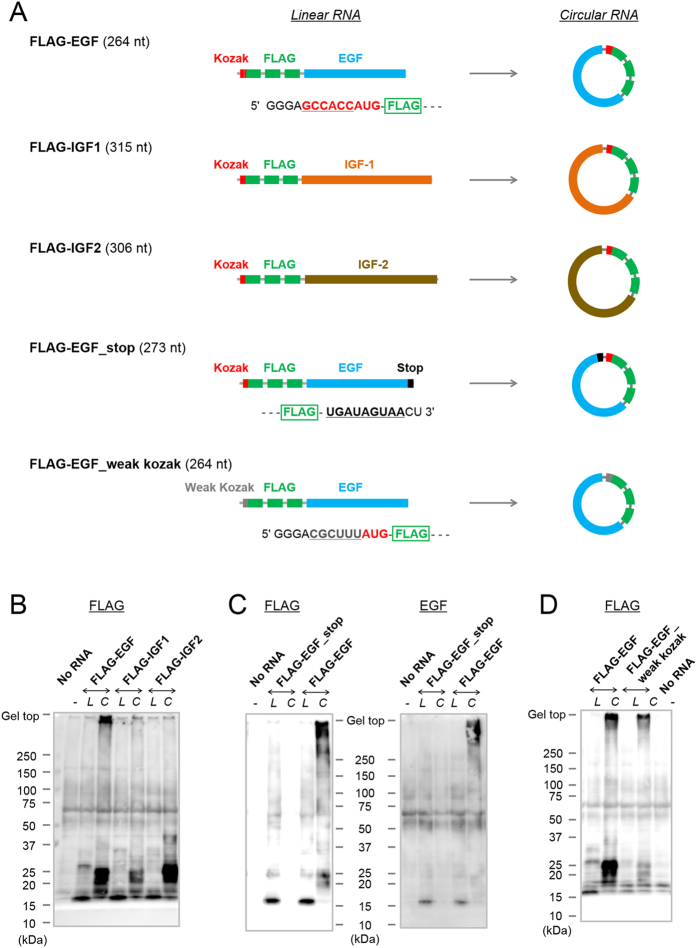
Synthesis of circular RNAs that encode human growth factors and their translation in rabbit reticulocyte lysate. (**A**) Schematic representation of the circular RNAs used in this study. The Kozak consensus sequence, the FLAG coding sequence and growth factor-coding sequences are indicated. The nucleotide sequences of the Kozak sequence and its mutant are shown. (**B**–**D**) Western blot analysis of the translation reaction in rabbit reticulocyte lysate. Circular RNAs and their linear precursors (2.4 μg in **B**, **C** and 1.2 μg in **D**) were incubated at 30 °C for 6 h in a 20 μL reaction. Anti-FLAG (**B**, left panel of **C** and **D**) or anti-EGF antibody (right panel of **C**) were used to detect the product. RNAs tested are indicated in the figure. Letters (**L**,**C**) in the figure denote linear or circular RNA, respectively.

To obtain some mechanistic insight into translation of circular RNAs, five different circular RNAs were synthesised (Fig. 6A, Supplementary Fig. S4, Supplementary Tables S3 and S4). Circular RNAs coding for human epidermal growth factor (EGF), insulin-like growth factor-1 (IGF-1) or insulin-like growth factor-2 (IGF-2), positioned just downstream of a Kozak sequence and FLAG-coding sequences were prepared, and their translation in RRL was compared (Fig. 6B; **FLAG-EGF**, **FLAG-IGF1** and **FLAG-IGF2**, respectively). Strong signal just below the top of the gel, which indicates that translation initiation had occurred and that the elongation reaction had cycled around the circle, was observed only when a circular RNA **FLAG-EGF** was tested (Fig. 6B). It is likely that the translation initiation occurred on the other two circular RNAs, **FLAG-IGF1** and **FLAG-IGF2**, because their translation products at around 20–25 kDa seem to be longer than those of the linear RNA (Fig. 6B). One possibility is that differences in secondary structures affected the translation reaction. The coding region for EGF contained the fewest GC nucleotides among the three (55% for EGF, 59% for IGF-1 and 66% for IGF-2, Supplementary Table S3)^26^,^27^. These circular RNAs produced peptides of high molecular weight in an *E. coli* cell-free translation system upon incubation, as well as in RRL (Supplementary Fig. S5)^1^. In the prokaryotic system, circular **FLAG-EGF** was also most efficiently translated among the three circular RNAs. As shown in Fig. 6C, no translation product was observed from circular RNA **FLAG-EGF_stop**, which contained stop codons, and thus a finite ORF in the sequence. The translation product from the circular **FLAG-EGF** was also detected using anti-EGF antibody, not only by anti-FLAG antibody (right panel of Fig. 6C). We then examined whether the strength of the Kozak consensus sequence has an effect on translation of circular RNA. The Kozak sequence within **FLAG-EGF** was mutated to a weaker, less-effective sequence (Fig. 6A and Supplementary Table S3, **FLAG-EGF_weak kozak**)^21^,^28^,^29^. As shown in Fig. 6D, the circular RNA with the weaker Kozak sequence also produced translation product just below the top of the gel. Decrease in the intensity of the product when compared with that from a circular RNA with the optimum Kozak sequence indicates that this mutation reduced the efficiency of translation initiation. As also seen in a prokaryotic system, however, when initiation has occurred once on a circular RNA with an infinite ORF, even if the initiation is ineffective, the elongation process can revolve around the circle multiple times producing high-molecular weight proteins^1^.

## Discussion

In eukaryotic translation systems, it was thought that circular mRNA can be translated only if it contains a sequence for internal ribosome entry^24^. However, in this study, we demonstrated that circular RNA can be translated without such particular sequences, even in living human cells. The translatable circular RNAs used in this study contained repeated FLAG-coding sequences. We can attribute the detection of the translation product to the highly sensitive FLAG system. However, more importantly, based on the result shown in Fig. 6C, the RCA mechanism was essential for the detection of translation initiation on circular RNA, which is usually quite inefficient. As for the importance of the Kozak consensus sequence for the initiation of translation on circular RNA, only a moderate reduction in the amount of translation product was observed when the optimal Kozak sequence was mutated to a weaker one. This result might indicate that the flanking sequences of an initiation codon do not strongly influence the translation of circular RNA by the RCA mechanism.

Recent studies have shown that circular RNAs composed of exonic sequences are abundant in human cells^14^,^15^,^30^,^31^. Previous knowledge of exonic circular RNA was limited to a handful of genes, and circular RNAs were thought to be an exceptional species produced by missplicing events^12^,^32^,^33^. However, high-throughput sequence analysis of the transcriptome has revealed that circular RNAs are conserved and more prevalent in eukaryotes than previously thought^14^,^15^,^30^,^31^. For instance, it has been reported that circular RNA molecules emanate from >14% of transcribed genes in human fibroblast cells^14^. Endogenous circular RNAs were shown to function as post-transcriptional regulators, for example as competitive inhibitors for microRNAs, known as microRNA sponges^15^,^31^. Our results indicate that circular RNA molecules can be translated into protein even without any IRES sequence, poly A or cap structure.

In conclusion, we demonstrated that circular RNA with an infinite ORF is a substrate for RCA of peptide in living human cells. Because endogenous circular RNAs are known to be abundant in human cells and may be translated into proteins, we are now examining whether endogenous circular RNAs can be translated in mammalian translation systems.

## Methods

### Denaturing polyacrylamide gel electrophoresis (PAGE) of RNA

Enzymatic reactions were analysed by denaturing PAGE [4–8% polyacrylamide (acrylamide/*N, N*′-methylenebisacrylamide, 19:1), 7.5 M urea, 25% (v/v) formamide, 89 mM Tris, 89 mM boric acid, 2 mM EDTA]. The RNA samples were mixed with equal amount of 2× formamide loading solution [80% (v/v) formamide, 10 mM EDTA pH 8.0, 0.1 mg/mL xylene cyanol FF, 0.1 mg/mL bromophenol blue], and heated at 90 °C for 3 min before being loaded on the gel. The gels were electrophoresed and stained with SYBR Green II (Lonza) and visualised on a BioRad ChemiDoc XRS+ System (BioRad, Hercules, CA, USA), Luminescent Image Analyser LAS 4000 (Fujifilm, Tokyo, Japan) or FAS-IV Imaging System (Nippon Genetics, Tokyo, Japan). Low Range ssRNA Ladder (New England Biolabs, Ipswich, MA, USA) was used as the size marker. RNAs were also purified by preparative denaturing PAGE. The RNA bands were visualised by UV shadowing, and crushed and extracted with water. The extracts were desalted by centrifugation with Amicon Ultra-0.5 mL or Amicon Ultra-4 mL centrifugal filter unit (Millipore, Billerica, MA, USA), according to the manufacturer’s instructions. The RNAs were then precipitated with sodium acetate (pH 5.2) and 2-propanol.

### Synthesis of linear RNAs by *in vitro* transcription

Linear RNAs were transcribed *in vitro* by T7 RNA polymerase from corresponding dsDNA templates (Supplementary Tables S2 and S4). The dsDNA templates for **4×**, **8×** and **12× FLAG** RNA were synthesised in our laboratory. The DNA templates for **FLAG-EGF**, **FLAG-IGF1**, **FLAG-IGF2** and **FLAG-EGF_weak kozak** were synthesised by Integrated DNA technologies, Inc (Coralville, IA, USA). The DNA sequence was amplified by PCR using PrimeSTAR HS DNA Polymerase (Takara Bio, Shiga, Japan: 0.01 ng/μL DNA vector, 1× PrimeSTAR buffer, 0.2 mM dNTPs, 1.5 μM primers, 0.025 units/μL polymerase)^34^. Linear RNA strands were prepared from the PCR products by *in vitro* transcription. Linear RNAs were prepared using the MEGAscript High Yield Transcription Kit (Invitrogen by Thermo Fisher Scientific, Waltham, MA, USA) except **4× FLAG** linear RNA, which was transcribed using T7 polymerase obtained from Takara Bio. 5′-mono-phosphorylated *in vitro* transcripts were obtained by adding an excess amount of GMP (7.5 mM) to GTP (1.5 mM) in the reaction mixture^7^. Based on the efficiency of the subsequent circularisation reaction, most of the transcription reaction appeared to be primed from GMP. After incubation, the reaction was treated with DNase to remove the DNA template. The transcribed RNAs were purified by preparative 5% denaturing PAGE.

### Circularisation of linear RNA using T4 RNA ligase 2

Transcribed linear RNA was circularised using T4 RNA ligase 2 (New England Biolabs) on a 20-nt DNA oligomer (guide DNA) as template (Supplementary Figs. S1 and S4). After annealing with a guide DNA (3–5 μM), 1 μM linear RNA was incubated with 0.0125 U/μL T4 RNA ligase 2 in a mixture of 50 mM Tris-HCl (pH 7.5), 2 mM MgCl_2_, 1 mM DTT and 0.4 mM ATP at 37 °C for 3 h. The ligated products were purified by preparative 5 or 6% denaturing PAGE.

### Circularity check of the RNA using RNase R

RNA (0.04 μg/μL) was incubated with RNase R (1 unit/μL; Epicenter, Madison, WI, USA) in 20 mM Tris-HCl (pH 8.0), 0.1 M KCl, and 0.1 mM MgCl_2_ at 37 °C for 10 min. The reaction mixture (5 μL) was analysed by 6% denaturing PAGE.

### Western blot analysis of the translation reaction of circular RNA in rabbit reticulocyte lysate

Circular RNA was incubated for 5 h or overnight in rabbit reticulocyte lysate (Promega, Fitchburg, WI, USA) at 30 °C in a volume of 20–25 μL. The final composition of the reaction mixture contained 70% rabbit reticulocyte lysate, 10 μM methionine and leucine, 20 μM amino acids other than methionine and leucine, 0.8 U/μL RNase inhibitor (Toyobo, Osaka, Japan). Aliquots (2.5 μL) were taken from the mixture and separated on 10–20% gradient polyacrylamide/sodium dodecyl sulfate (SDS) gels (Atto, Tokyo, Japan). As for the experiments shown in Fig. 6, acetic acid (0.32 μL) and water (300 μL) were added subsequently to the reaction mixture (16 μL) and it was centrifuged at 20,817× g for 10 min at 15 °C. The supernatant was removed and the pellet was dissolved in 30 μL of 2× SDS sample buffer (0.125 M Tris-HCl, pH 6.8, 4% SDS, 30% glycerol, 5% 2-mercaptoethanol, 0.01% bromophenol blue) at 70 °C for 15 min. The majority of the hemoglobin protein was removed during this process whereas proteins other than hemoglobin were concentrated (this purification procedure was provided by the technical service division of Promega). After centrifugation at 1,400× g for 5 min, the supernatant (10 μL) was analysed on 10–20% gradient polyacrylamide/ SDS gels. The Precision Plus Protein Kaleidoscope Standard (BioRad) was used as the size marker. After being electrotransferred to a polyvinylidene fluoride (PVDF) membrane (Millipore) using the semi-dry method^35^, the blot was incubated with an anti-FLAG M2 monoclonal antibody (Sigma-Aldrich, St. Louis, MO, USA) and anti-mouse IgG peroxidase conjugate (Sigma-Aldrich). Anti-EGF, anti-IGF1, anti-IGF2 were also used as the primary antibodies (Santa Cruz Biotech.) The blot was visualised using the SuperSignal West Femto Maximum Sensitivity Substrate Kit (Thermo Fisher Scientific, Waltham, MA, USA) on a Light-Capture cooled CCD camera system (Atto) or on a Luminescent Image Analyser LAS 4000 (Fujifilm).

### Western blot analysis of the translation reaction in cultured human cells

HeLa cells (RIKEN Cell Bank, Ibaraki, Japan) were grown in Dulbecco’s modified Eagle’s medium (DMEM; Invitrogen) supplemented with 10% fetal bovine serum (FBS; Invitrogen) at 37 °C under 5% CO_2_. Cells were passaged regularly to maintain exponential growth. Lipofectamine RNAiMAX (2 μL; Invitrogen) was added to a mixture of 1 μg RNA and 145 μL Opti-MEM I solution (Invitrogen) in a well of a 12-well plate. After incubation at room temperature for 15 min, 8.5 × 10^4^ HeLa cells suspended in DMEM containing 10% FBS (0.85 mL) were added to the RNA solution. After incubation for 24 h at 37 °C, the cells were collected and lysed with 0.2 mL of 1× Cell Lysis Buffer (Cell Signaling Technology, Danvers, MA, USA) containing 100 mM phenylmethylsulfonyl fluoride (Thermo Fisher Scientific). The lysates (7.5 μL) were subjected to SDS-PAGE (10–20% gradient gel, Atto) and western blot analysis as described above.

### Immunocytochemistry of the translation products in cultured human cells

HeLa cells were grown in DMEM supplemented with 10% FBS at 37 °C under 5% CO_2_. Cells were passaged regularly to maintain exponential growth. One day before transfection, cells were inoculated in a 24-well plate with a glass coverslip at the bottom of the wells. RNA (1 μg) was transfected into the cells using the transfection reagent, Oligofectamine (Invitrogen), according to the manufacturer’s instructions. After incubation for 24 h at 37 °C, the cells were fixed using 4% paraformaldehyde in phosphate-buffered saline (PBS) at room temperature for 30 min and then incubated with 0.3% Triton X-100 in PBS. After blocking with 1% bovine serum albumin (BSA) in PBS, the cells were incubated with 2 μg/mL of anti-FLAG M2 monoclonal antibody (Sigma-Aldrich) in PBS containing 1% BSA for 1 h. The cells were washed twice with PBS containing 0.3% Triton X-100 and then incubated with a mixture of Alexa Fluor 488-labelled anti mouse IgG antibody (Invitrogen) and 4′,6-diamidino-2-phenylindole (DAPI; 250 ng/mL) for 30 min. The cells were mounted on a glass slide using PermaFluor aqueous mounting medium (Thermo Fisher Scientific) and examined with an inverted Olympus IX-81 fluorescence microscope (Tokyo, Japan) equipped with an oil-immersion 60× objective (1.35 N.A.).

### Analysis of putative IRES activity in the FLAG-repeating sequence using a bicistronic reporter assay system

The EMCV-IRES-containing bicistronic vector, pβGal-IRES-CAT, was constructed by inserting the β-galactosidase (β-gal) gene from pCMV-LacZ Vector (Clontech, Mountain View, CA, USA) and the chloramphenicol acetyltransferase (CAT) gene from pCAT3-Basic Vector (Promega) into multi-cloning sites A and B of pIRES (Clontech), respectively. The control vector, pβGal-CAT, was obtained by removing the IRES region from the pβGal-IRES-CAT by inverse PCR. The FLAG repeat-containing vector, pβGal-4× FLAG-CAT, was constructed by inserting the 4× FLAG fragment (obtained from a vector which was synthesised at Integrated DNA Technologies Inc.) into the linker region of pβGal-CAT. The expression of the bicistronic mRNAs was driven by the cytomegalovirus (CMV) promoter. Nucleic acid sequences of vector inserts were confirmed by sequencing. HeLa cells were plated in a 6-well plate (4 × 10^5^ cells/well, 2 mL) one day before transfection. The cells were transfected with these plasmids (2.5 μg each/well) using Lipofectamine 2000 (Invitrogen) according to the manufacturer’s instructions. Twenty-four hours after transfection the cells were harvested and assessed for expression of β-gal and CAT by enzyme-linked immunosorbent assays (ELISA) using β-Gal ELISA and CAT ELISA kits (Roche Applied Science, Mannheim, Germany), respectively.

## Additional Information

**How to cite this article**: Abe, N. *et al*.Rolling Circle Translation of Circular RNA in Living Human Cells. *Sci. Rep*.**5**, 16435; doi: 10.1038/srep16435 (2015).

## Supplementary Material

Supplementary Information

## Figures and Tables

**Figure 1:**
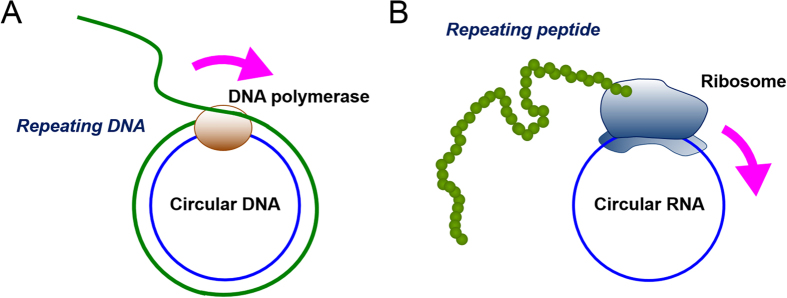
Rolling circle amplification of DNA (A) or peptide (B) on a small circular template.
