# Supplementary material for: Rolling Circle Translation of Circular RNA in Living Human Cells
Source: Sci Rep. 2015 Nov 10;5:16435. doi: 10.1038/srep16435 (PMC4639774; doi:10.1038/srep16435)
Supplement: Supplementary Information [file srep16435-s1.pdf]

Supplementary Information

Rolling Circle Translation of Circular RNA in Living Human Cells

Naoko Abe[1,2,3], Ken Matsumoto[5], Mizuki Nishihara[3], Yukiko Nakano[2,3], Aya Shibata[3], Hideto Maruyama[2,3], Satoshi Shuto[2], Akira Matsuda[2], Minoru Yoshida[5], Yoshihiro Ito[3,6], and Hiroshi Abe[1,2,3,4,*]

[1]Department of Chemistry, Graduate School of Science, Nagoya University, Furo-cho, Chikusa-ku, Nagoya 464-8602, Japan

[2]Faculty of Pharmaceutical Sciences, Hokkaido University, Kita-12, Nishi-6, Kita-ku, Sapporo 060-0812, Japan

[3]Nano Medical Engineering laboratory, RIKEN, 2-1 Hirosawa, Wako-Shi, Saitama 351-0198, Japan

[4]PRESTO, Japan Science and Technology Agency, 4-1-8 Honcho, Kawaguchi, Saitama 332-0012, Japan

[5]Chemical Genetics Laboratory, RIKEN, 2-1 Hirosawa, Wako-Shi, Saitama 351-0198, Japan

[6]Emergent Bioengineering Materials Research Team, RIKEN Center for Emergent Matter Science, 2-1 Hirosawa, Wako, Saitama 351-0198, Japan

[*]h-abe@chem.nagoya-u.ac.jp

Table of Contents

**Table S1:**	Sequence of RNA transcripts containing multiple FLAG coding sequences.
**Table S2:**	Sequences of chemically synthesized DNAs for the synthesis of circular RNAs containing multiple FLAG coding sequences by in vitro transcription.
**Table S3:**	Sequences of RNA transcripts that code a human growth factor.
**Table S4:**	Template dsDNA sequences for *in vitro* transcription to synthesize RNAs that code a human growth factor.
**Figure S1:**	Denaturing PAGE analysis of ligation reactions in the preparation of circular RNAs containing multiple FLAG-coding sequences.
**Figure S2:**	Western blot analysis of the translation product of linear RNAs containing multiple FLAG code after purification.
**Figure S3:**	Immunofluorescent staining of HeLa cells after the transfection of a circular RNA.
**Figure S4:**	Denaturing PAGE analysis of *in vitro* transcribed RNAs and subsequent circularization reactions in the preparation of circular RNAs coding human growth factors.
**Figure S5:**	Circular RNAs which code human growth factors were translated into peptides of high-molecular weight in an *E. coli* cell-free expression system.

Table S1. Sequence of RNA transcripts containing multiple FLAG coding sequences. The first and the last nucleotides are connected in the circularized molecule.

Name	Sequence (5′ to 3′)*
**4× FLAG** RNA (129 nt)	1 GGGAGCCACC AUGGACUACA AGGACGACGA CGACAAGAUC AUCGACUACA<br>51 AGGACGACGA CGACAAGAUC AUCGACUACA AGGACGACGA CGACAAGAUA<br>101 AUUGACUAUA AAGACGACGA CGAUAAAGU
**8× FLAG** RNA (258 nt)	1 GGGAGCCACC AUGGACUACA AGGACGACGA CGACAAGAUC AUCGACUACA<br>51 AGGACGACGA CGACAAGAUC AUCGACUACA AGGACGACGA CGACAAGAUA<br>101 AUUGACUAUA AAGACGACGA CGAUAAAGUA CUGGACUAUA AAGACGACGA<br>151 CGACAAGAUC AUCGACUACA AGGACGACGA CGACAAGAUC AUCGACUACA<br>201 AGGACGACGA CGACAAGAUU AUAGACUACA AAGACGACGA CGAUAAAUUC<br>251 AACCAGAA
**12× FLAG** RNA (387 nt)	1 GGGAGCCACC AUGGACUACA AGGACGACGA CGACAAGAUC AUCGACUACA<br>51 AGGACGACGA CGACAAGAUC AUCGACUACA AGGACGACGA CGACAAGAUA<br>101 AUUGACUAUA AAGACGACGA CGAUAAAGUA CUGGACUAUA AAGACGACGA<br>151 CGACAAGAUC AUCGACUACA AGGACGACGA CGACAAGAUC AUCGACUACA<br>201 AGGACGACGA CGACAAGAUU AUAGACUACA AAGACGACGA CGAUAAAUUC<br>251 AACCAGAUAU CCGAUUAUAA GGACGACGAC GACAAGAUCA UCGACUACAA<br>301 GGACGACGAC GACAAGAUCA UCGACUACAA GGACGACGAC GACAAGAUAA<br>351 UAGAUUACAA GGACGACGAC GAUAAACCCU CUAGAGG

* The Kozak sequence is underlined. FLAG coding sequences have gray-colored background.

Table S2. Template dsDNA sequences for *in vitro* transcription to synthesize RNAs containing multiple FLAG coding sequences.

Name	Sequence (5′ to 3′)*
4× **FLAG** DNA<br>(155 bp)	CGCGGATCCTAATACGACTCACTATAGGGA*GCCACCATG*___G___**ACTACAAGGACGACGACGACAA**<br>**G**ATCATC**GACTACAAGGACGACGACGACAAG**ATCATC**GACTACAAGGACGACGACGACAAG**A<br>TAATT**GACTATAAAGACGACGACGATAAA**GT
8× **FLAG** DNA<br>(284 bp)	CGCGGATCCTAATACGACTCACTATAGGGA*GCCACCATG*___G___**ACTACAAGGACGACGACGACAA**<br>**G**ATCATC**GACTACAAGGACGACGACGACAAG**ATCATC**GACTACAAGGACGACGACGACAAG**A<br>TAATT**GACTATAAAGACGACGACGATAAA**GTACTG**GACTATAAAGACGACGACGACAAG**ATC<br>ATC**GACTACAAGGACGACGACGACAAG**ATCATC**GACTACAAGGACGACGACGACAAG**ATTAT<br>A**GACTACAAAGACGACGACGATAAA**TTCAACCAGAA
12× **FLAG** DNA<br>(413 bp)	CGCGGATCCTAATACGACTCACTATAGGGA*GCCACCATG*___G___**ACTACAAGGACGACGACGACAA**<br>**G**ATCATC**GACTACAAGGACGACGACGACAAG**ATCATC**GACTACAAGGACGACGACGACAAG**A<br>TAATT**GACTATAAAGACGACGACGATAAA**GTACTG**GACTATAAAGACGACGACGACAAG**ATC<br>ATC**GACTACAAGGACGACGACGACAAG**ATCATC**GACTACAAGGACGACGACGACAAG**ATTAT<br>A**GACTACAAAGACGACGACGATAAA**TTCAACCAGATATCC**GATTATAAGGACGACGACGACA**<br>**AG**ATCATC**GACTACAAGGACGACGACGACAAG**ATCATC**GACTACAAGGACGACGACGACAAG**<br>ATAATA**GATTACAAGGACGACGACGATAAA**CCCTCTAGAGG

* T7 promoter sequence is shown in a box with a solid line. The G base highlighted in yellow is the first base incorporated into RNA during transcription. The Kozak sequence is witten in italic and underlined. FLAG-coding sequences are shown in bold letters.

Table S3. Sequences of RNA transcripts that code a human growth factor. The corresponding amino acid sequences are shown under the nucleotides in bold letters. Molecular masses of expected translation product are shown in the left column.

Name of RNA	RNA sequence (5′ to 3′) and corresponding amino acid sequence*
**FLAG-EGF**<br>(264 nt)<br><br>From linear RNA,<br>9,862.8 Da<br>From circular RNA,<br>10,205.2 Da/ round	G GGA GCC ACC AUG GAC UAC AAG GAC GAC GAC GAC AAG AUC AUC GAC UAU<br>***G A T*** **M D Y K D D D D K I I D Y**<br>AAA GAC GAC GAC GAU AAA GGU GGC GAC UAU AAG GAC GAC GAC GAC AAA<br>**K D D D D K G G D Y K D D D D K**<br>GCC AUU AAU AGU GAC UCU GAG UGU CCC CUG UCC CAC GAC GGG UAC UGC<br>**A I N S D S E C P L S H D G Y C**<br>CUC CAC GAC GGU GUG UGC AUG UAU AUU GAA GCA UUG GAC AAG UAC GCC<br>**L H D G V C M Y I E A L D K Y A**<br>UGC AAC UGU GUU GUU GGC UAC AUC GGG GAG CGC UGU CAG UAC CGA GAC<br>**C N C V V G Y I G E R C Q Y R D**<br>CUG AAG UGG UGG GAA CUG CGC CU-<br>**L K W W E L R** ***L***
**FLAG-IGF1**<br>(315 nt)<br><br>From linear RNA,<br>12,004.3 Da<br>From circular RNA,<br>12,346.6 Da/ round	G GGA GCC ACC AUG GAC UAC AAG GAC GAC GAC GAC AAG AUC AUC GAC UAU<br>***G A T*** **M D Y K D D D D K I I D Y**<br>AAA GAC GAC GAC GAU AAA GGU GGC GAC UAU AAG GAC GAC GAC GAC AAA<br>**K D D D D K G G D Y K D D D D K**<br>GCC AUU GGA CCG GAG ACG CUC UGC GGG GCU GAG CUG GUG GAU GCU CUU<br>**A I G P E T L C G A E L V D A L**<br>CAG UUC GUG UGU GGA GAC AGG GGC UUU UAU UUC AAC AAG CCC ACA GGG<br>**Q F V C G D R G F Y F N K P T G**<br>UAU GGC UCC AGC AGU CGG AGG GCG CCU CAG ACA GGU AUG GUG GAU GAG<br>**Y G S S S R R A P Q T G M V D E**<br>UGC UGC UUC CGG AGC UGU GAU CUA AGG AGG CUG GAG AUG UAU UGC GCA<br>**C C F R S C D L R R L E M Y C A**<br>CCC CUC AAG CCU GCC AAG UCA GCU CU-<br>**P L K P A K S A** ***L***
**FLAG-IGF2**<br>(306 nt)<br><br>From linear RNA,<br>11,287.4 Da<br>From circular RNA,<br>11,629.8 Da/ round	G GGA GCC ACC AUG GAC UAC AAG GAC GAC GAC GAC AAG AUC AUC GAC UAU<br>***G A T*** **M D Y K D D D D K I I D Y**<br>AAA GAC GAC GAC GAU AAA GGU GGC GAC UAU AAG GAC GAC GAC GAC AAA<br>**K D D D D K G G D Y K D D D D K**<br>GCC AUU GCU UAC CGC CCC AGU GAG ACC CUG UGC GGC GGG GAG CUG GUG<br>**A I A Y R P S E T L C G G E L V**<br>GAC ACC CUC CAG UUC GUC UGU GGG GAC CGC GGC UUC UAC UUC AGC AGG-<br>**D T L Q F V C G D R G F Y F S R**<br>CCC GCA AGC CGU GUG AGC CGU CGC AGC CCU GGC AUC GUU GAG GAG UGC<br>**P A S R V S R R S P G I V E E C**<br>UGU UUC CGC AGC UGU GAC CUG GCC CUC CUG GAG ACG UAC UGU GCU ACC<br>**C F R S C D L A L L E T Y C A T**<br>CCC GCC AAG UCC GAG CU-<br>**P A K S E** ***L***
**FLAG-EGF_stop**<br>(273 bp)<br><br>From linear RNA,<br>9,862.8 Da<br>From circular RNA,<br>9,862.8 Da	G GGA GCC ACC AUG GAC UAC AAG GAC GAC GAC GAC AAG AUC AUC GAC UAU<br>**M D Y K D D D D K I I D Y**<br>AAA GAC GAC GAC GAU AAA GGU GGC GAC UAU AAG GAC GAC GAC GAC AAA<br>**K D D D D K G G D Y K D D D D K**<br>GCC AUU AAU AGU GAC UCU GAG UGU CCC CUG UCC CAC GAC GGG UAC UGC<br>**A I N S D S E C P L S H D G Y C**<br>CUC CAC GAC GGU GUG UGC AUG UAU AUU GAA GCA UUG GAC AAG UAC GCC<br>**L H D G V C M Y I E A L D K Y A**<br>UGC AAC UGU GUU GUU GGC UAC AUC GGG GAG CGC UGU CAG UAC CGA GAC<br>**C N C V V G Y I G E R C Q Y R D**<br>CUG AAG UGG UGG GAA CUG CGC **UGA UAG UAA** CU-<br>**L K W W E L R**

**FLAG-EGF_weak**	G GGA CGC UUU AUG GAC UAC AAG GAC GAC GAC GAC AAG AUC AUC GAC UAU
**kozak**	***G*** ***R*** ***F*** **M** **D** **Y** **K** **D** **D** **D** **D** **K** **I** **I** **D** **Y**
(264 bp)	AAA GAC GAC GAC GAU AAA GGU GGC GAC UAU AAG GAC GAC GAC GAC AAA
	**K** **D** **D** **D** **D** **K** **G** **G** **D** **Y** **K** **D** **D** **D** **D** **K**
	GCC AUU AAU AGU GAC UCU GAG UGU CCC CUG UCC CAC GAC GGG UAC UGC
	**A** **I** **N** **S** **D** **S** **E** **C** **P** **L** **S** **H** **D** **G** **Y** **C**
From linear RNA,	CUC CAC GAC GGU GUG UGC AUG UAU AUU GAA GCA UUG GAC AAG UAC GCC
10,323.3 Da	**L** **H** **D** **G** **V** **C** **M** **Y** **I** **E** **A** **L** **D** **K** **Y** **A**
From circular RNA,	UGC AAC UGU GUU GUU GGC UAC AUC GGG GAG CGC UGU CAG UAC CGA GAC
10,796.8 Da/ round	**C** **N** **C** **V** **V** **G** **Y** **I** **G** **E** **R** **C** **Q** **Y** **R** **D**
	CUG AAG UGG UGG GAA CUG CGC CU-
	**L** **K** **W** **W** **E** **L** **R** ***L***

*Authentic Kozak sequences are underlined. A start codon methionine for linear RNA is shown in red. In-frame stop codons are shown in bold text. Amino acids which would be translated only on circular RNA are shown in italic.

Table S4. Template dsDNA sequences for *in vitro* transcription to synthesize RNAs that code a human growth factor. Sense sequences are shown in this table.

Name	Sequence (5′ to 3′)*
**FLAG-EGF****<br>(290 bp)	CGCGGATCCTAATACGACTCACTATAGGGAGCCACCATGGACTACAAGGACGACGACGACAA<br>GATCATCGACTATAAAGACGACGACGATAAAGGTGGCGACTATAAGGACGACGACGACAAAG<br>CCATTAATAGTGACTCTGAGTGTCCCCTGTCCCACGACGGGTACTGCCTCCACGACGGTGTG<br>TGCATGTATATTGAAGCATTGGACAAGTACGCCTGCAACTGTGTTGTTGGCTACATCGGGGA<br>GCGCTGTCAGTACCGAGACCTGAAGTGGTGGGAACTGCGCCT
**FLAG-IGF1*****<br>(341 bp)	CGCGGATCCTAATACGACTCACTATAGGGAGCCACCATGGACTACAAGGACGACGACGACAA<br>GATCATCGACTATAAAGACGACGACGATAAAGGTGGCGACTATAAGGACGACGACGACAAAG<br>CCATTGGACCGGAGACGCTCTGCGGGGCTGAGCTGGTGGATGCTCTTCAGTTCGTGTGTGGA<br>GACAGGGGCTTTTATTTCAACAAGCCCACAGGGTATGGCTCCAGCAGTCGGAGGGCGCCTCA<br>GACAGGTATGGTGGATGAGTGCTGCTTCCGGAGCTGTGATCTAAGGAGGCTGGAGATGTATT<br>GCGCACCCCTCAAGCCTGCCAAGTCAGCTCT
**FLAG-IGF2******<br>(332 bp)	CGCGGATCCTAATACGACTCACTATAGGGAGCCACCATGGACTACAAGGACGACGACGACAA<br>GATCATCGACTATAAAGACGACGACGATAAAGGTGGCGACTATAAGGACGACGACGACAAAG<br>CCATTGCTTACCGCCCCAGTGAGACCCTGTGCGGCGGGGAGCTGGTGGACACCCTCCAGTTC<br>GTCTGTGGGGACCGCGGCTTCTACTTCAGCAGGCCCGCAAGCCGTGTGAGCCGTCGCAGCCC<br>TGGCATCGTTGAGGAGTGCTGTTTCCGCAGCTGTGACCTGGCCCTCCTGGAGACGTACTGTG<br>CTACCCCCGCCAAGTCCGAGCT
**FLAG-EGF_stop****<br>(299 bp)	CGCGGATCCTAATACGACTCACTATAGGGAGCCACCATGGACTACAAGGACGACGACGACAA<br>GATCATCGACTATAAAGACGACGACGATAAAGGTGGCGACTATAAGGACGACGACGACAAAG<br>CCATTAATAGTGACTCTGAGTGTCCCCTGTCCCACGACGGGTACTGCCTCCACGACGGTGTG<br>TGCATGTATATTGAAGCATTGGACAAGTACGCCTGCAACTGTGTTGTTGGCTACATCGGGGA<br>GCGCTGTCAGTACCGAGACCTGAAGTGGTGGGAACTGCGC**TGATAGTAA**CT
**FLAG-EGF_weak kozak****<br>(290 bp)	CGCGGATCCTAATACGACTCACTATAGGGACGCTTTATGGACTACAAGGACGACGACGACAA<br>GATCATCGACTATAAAGACGACGACGATAAAGGTGGCGACTATAAGGACGACGACGACAAAG<br>CCATTAATAGTGACTCTGAGTGTCCCCTGTCCCACGACGGGTACTGCCTCCACGACGGTGTG<br>TGCATGTATATTGAAGCATTGGACAAGTACGCCTGCAACTGTGTTGTTGGCTACATCGGGGA<br>GCGCTGTCAGTACCGAGACCTGAAGTGGTGGGAACTGCGCCT

* T7 promoter sequence is shown in a box with a solid line. The G base highlighted in yellow is the first base incorporated into RNA during transcription. The Kozak sequence is witten in italic and underlined. FLAG-coding sequences are shown in bold letters. Coding sequences for EGF, IGF-1 and IGF-2 have gray-colored background.

** GenBank: X04571.1

*** GenBank: A29117.1

**** GenBank: EU622024.1

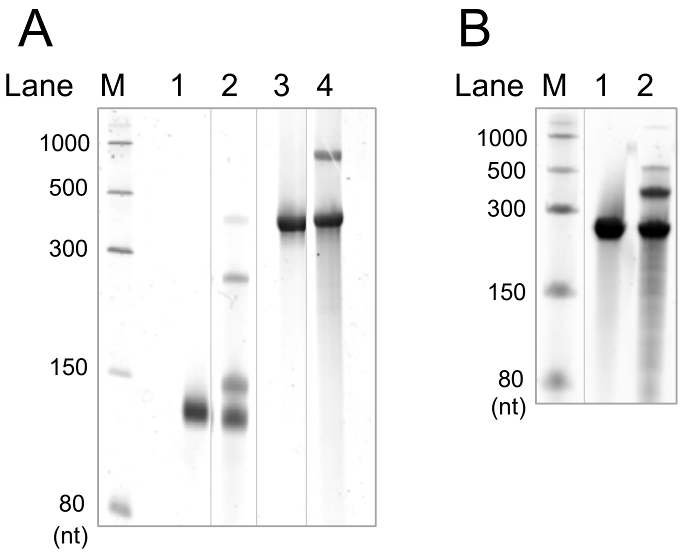

Figure S1. Denaturing PAGE analysis of ligation reactions in the preparation of circular RNAs containing multiple FLAG-coding sequences. The gel contained 5% polyacrylamide. The gels were visualized by SYBR Green II staining. (A) Lane 1, **4× FLAG** linear RNA; lane 2, **4× FLAG** linear RNA + guide DNA + T4 RNA ligase 2; lane 3, **12× FLAG** linear RNA; lane 4, **12× FLAG** linear RNA + T4 RNA Ligase 2. (B) Lane 1, **8× FLAG** linear RNA; lane 2, **8× FLAG** linear RNA + guide DNA + T4 RNA ligase 2.

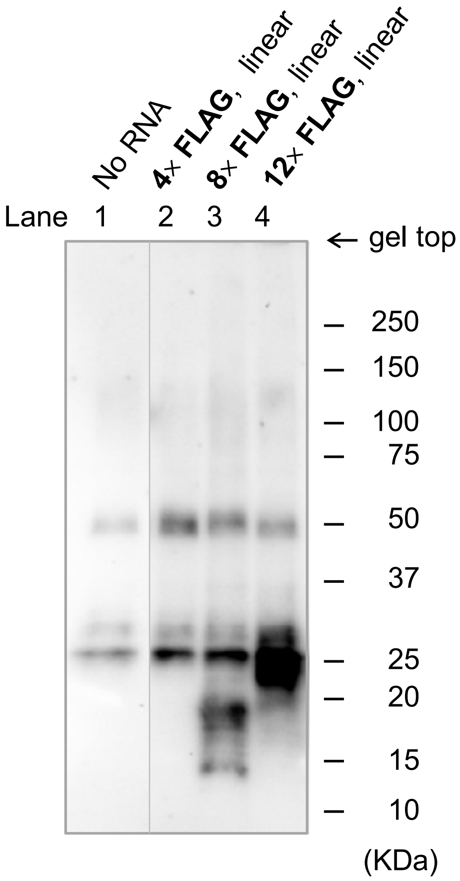

Figure S2. Western blot analysis of the translation product of linear RNAs after purification. RNA (0.48 μM) was incubated in rabbit reticulocyte lysate at 30°C for overnight. After incubation, the sample was purified using anti-FLAG antibody-conjugated agarose beads according to the manufacturer's protocol (Wako Pure Chemicals). The anti-FLAG antibody dissociated from the beads was detected at around 25 and 50 KDa of the size marker.

Expected sequences of translated peptides and their molecular mass are shown below.

From **4× FLAG** linear RNA:

MDYKDDDDKI IDYKDDDDKI IDYKDDDDKI IDYKDDDDK (39 a. a.)

Molecular mass: 4,808.0

From **8× FLAG** linear RNA:

MDYKDDDDKI IDYKDDDDKI IDYKDDDDKI IDYKDDDDKV LDYKDDDDKI IDYKDDDDKI IDYKDDDDKI IDYKDDDDKF NQ (82 a. a.)

Molecular mass: 10,068.6

From **12× FLAG** linear RNA:

MetDYKDDDDKI IDYKDDDDKI IDYKDDDDKI IDYKDDDDKV LDYKDDDDKI IDYKDDDDKI IDYKDDDDKI IDYKDDDDKF NQISDYKDDD DKIIDYKDDD DKIIDYKDDD DKIIDYKDDD DKPSR (125 a. a.)

Molecular mass: 15,268.0

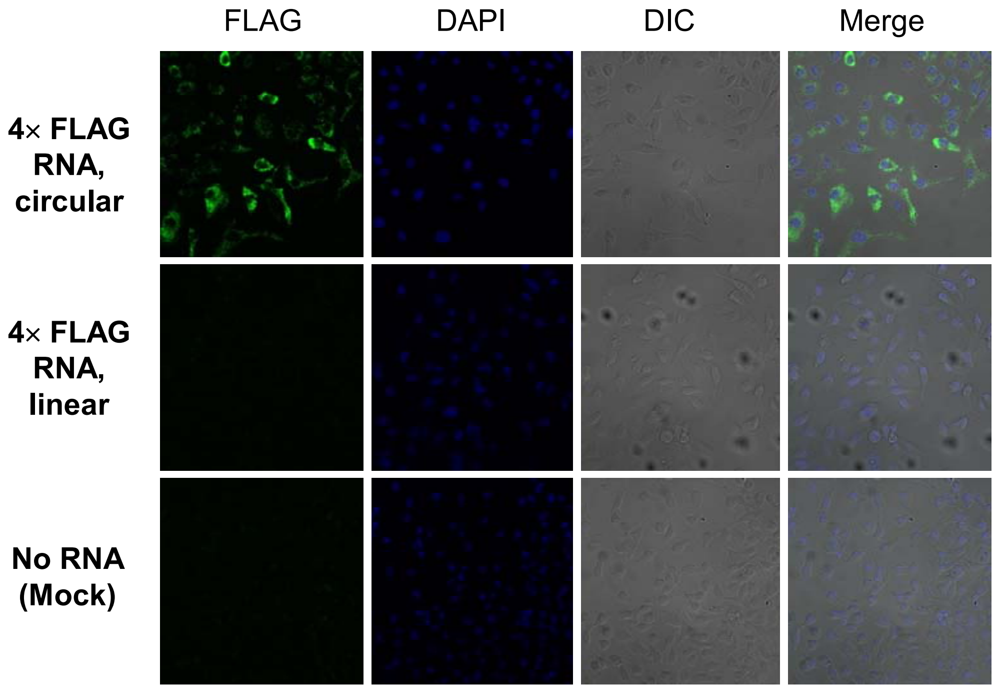

Figure S3. Immunofluorescent staining of HeLa cells after the transfection of a circular RNA named **4× FLAG**. Microscopic imaging of the translation product was performed using anti-FLAG antibody labeled with Alexa Fluor 488 (green). Cells were counterstained with DAPI (4',6-diamidino-2-phenylindole; blue) to image nuclei. Differential interference contrast (DIC) images and the merged image of the three (Merge) are also shown. The 129 nt RNA sequence was chemically synthesized on a DNA/RNA synthesizer with 5′-phosphorylation. After being purified, the linear RNA was circularized using T4 RNA ligase 2 on a short complementary DNA oligo.

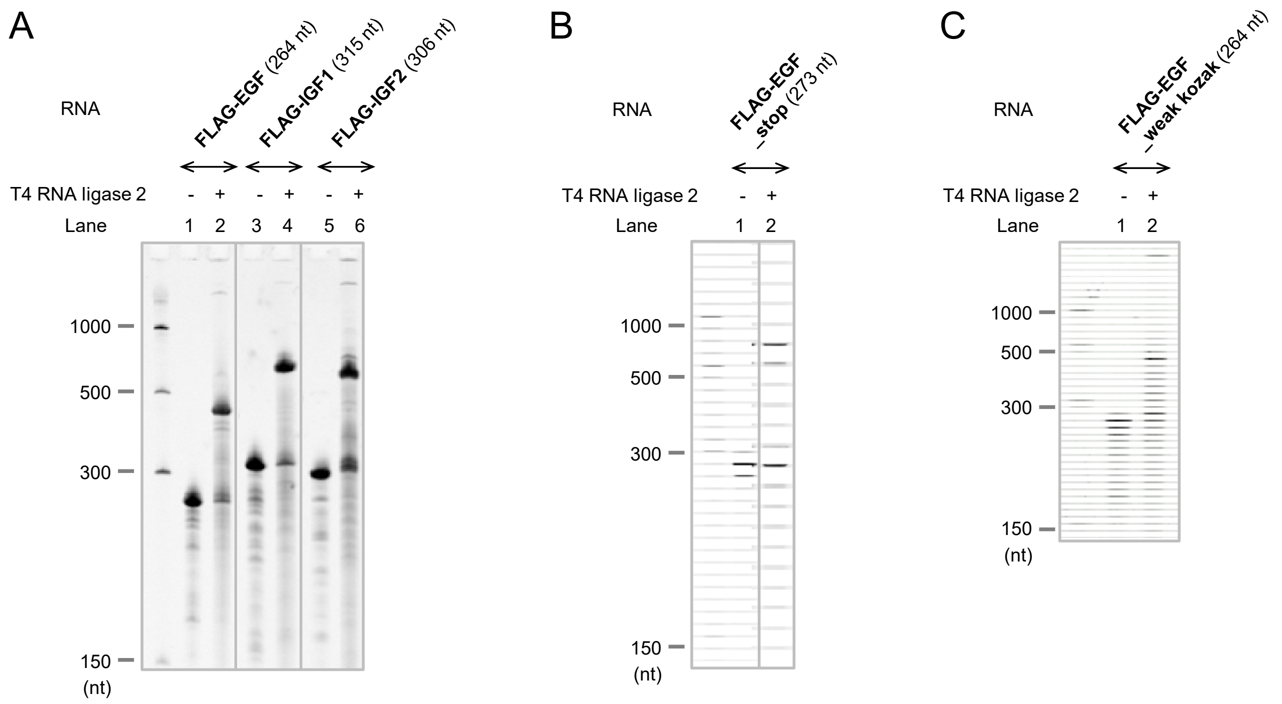

Figure S4. Denaturing PAGE (6%) analysis of *in vitro* transcribed RNAs and subsequent circularization reactions in the preparation of circular RNAs coding a human growth factor. The RNAs were annealed on a complementary 20-nt DNA oligomer and incubated with T4 RNA ligase 2 at 37°C for 4 h. The gels were visualized by SYBR Green II staining. (A) Lane 1, **FLAG-EGF** transcribed linear RNA; lane 2, **FLAG-EGF** transcribed linear RNA + ligase; lane 3, **FLAG-IGF1** transcribed linear RNA; lane 4, **FLAG-IGF1** transcribed linear RNA + ligase; lane 5, **FLAG-IGF2** transcribed linear RNA; lane 6, **FLAG-IGF2** transcribed linear RNA + ligase. (B) Lane 1, **FLAG-EGF_stop** transcribed linear RNA; lane 2, **FLAG-EGF_stop** transcribed linear RNA + ligase. (C) Lane 1, **FLAG-EGF_weak kozak** transcribed linear RNA; lane 2, **FLAG-EGF_weak kozak** transcribed linear RNA + ligase.

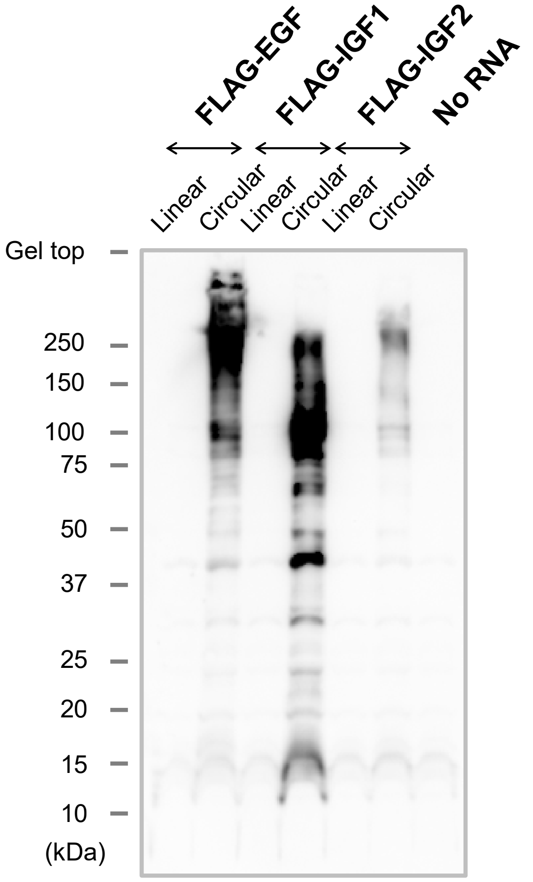

Figure S5. Circular RNAs which code a human growth factor were translated into peptides of high-molecular weight in an E. coli cell-free expression system. The RNA was translated in a cell-free translation system (PURExpress, New England Biolabs) at 1 μM concentration. After being incubated at 37°C for 2 h, 1 μL of each reaction mixture was analyzed by SDS-PAGE using a 10–20% Tris-glycine gel. The peptides were then transferred to a PVDF membrane. Anti-FLAG M2 monoclonal antibody (Sigma-Aldrich) and anti-mouse IgG peroxidase-conjugated antibody (Sigma-Aldrich) were used to visualize the blot. Letters "L" and "C" in the figure stand for linear RNA or circular RNA, respectively.
